# Effectiveness and Safety of Dietetic Supplementation of a New Nutraceutical on Lipid Profile and Serum Inflammation Biomarkers in Hypercholesterolemic Patients

**DOI:** 10.3390/molecules23051168

**Published:** 2018-05-14

**Authors:** Graziano Riccioni, Maria Alessandra Gammone, Walter Currenti, Nicolantonio D’Orazio

**Affiliations:** 1 Cardiology Unit, San Camillo de Lellis Hospital, Manfredonia, Foggia 71100, Italy; 2 Human Nutrition, “G. D’Annunzio” University, Chieti-Pescara 66013, Italy; m.alessandra.gammone@gmail.com; 3 Department of Biomedical and Biotechnological Science, School of Medicine, University of Catania, Catania 95100, Italy; walter.currenti@unich.it (W.C.); ndorazio@unich.it (N.D.)

**Keywords:** berberine, red yeast rice, monacolin K, resveratrol, lycopene, cholesterol, triglycerides, hypercholesterolemia, hydroxytyrosol, zinc

## Abstract

*Background*: To assess the effectiveness and safety of a new nutraceutical (NC) on lipid profile, inflammation biomarkers and creatine phosphokinase (CPK) serum levels in hypercholesterolemic patients. *Methods*: 40 patients underwent hypolipemic treatment with NC. Initial and final (after 12 weeks) screening included medical history, physical examination, and measurement of serum lipid profile (total cholesterol, HDL-cholesterol, LDL-cholesterol, and triglycerides), hepatic (GOT, GPT, γGT), and renal (serum creatinine and urea) functions, CPK level and many inflammation biomarkers (hs-CRP and fibrinogen). At the screening visit, all patients were instructed to follow a normocaloric and hypolipidic diet during the study period. *Results*: The treatment with supplementation of NC demonstrated a significant reduction of serum total cholesterol (224 ± 11.2 mg/dL vs. 178 ± 10.7; *p* < 0.001), LDL-cholesterol (141 ± 10.6 vs. 116 + 10.1; *p* < 0.001), triglycerides (183 ± 13 vs. 159 ± 11.5; *p* < 0.01), serum inflammatory biomarkers as hs-CRP (2.24 ± 0.83 vs. 1.76 ± 0.61 mg/dL; *p* < 0.01), fibrinogen (315 ± 43 vs. 199 ± 41 mg/dL; *p* < 0.01) and a significantly increase of HDL-cholesterol (44 ± 7 vs. 53 ± 7 mg/dL; *p* < 0.01). Hepatic and renal function and serum CPK were normal. No adverse events was reported. *Conclusions*: The treatment with NC has demonstrated a significant reduction of LDL-cholesterol (−17.73%), total cholesterol (−20.53%) and triglycerides (−13.1%), with a significant increase of HDL-cholesterol values (+20.45%). The improvement of lipid profile was associated with a significant reduction of serum inflammation biomarkers as hs-PCR (−27%) and fibrinogen (−36.8%) with good tolerability profile.

## 1. Introduction

Cardiovascular diseases (CVD) represent one of the most important cause of mortality and morbidity in the industrialized world [1,2]. The atherosclerotic process is an important pathogenetic factor of CVD; in particular, hypercholesterolemia is one of most important risk factors that contribute to the formation and progression of atherosclerotic plaque secondary to elevated levels of low density lipoprotein cholesterol (LDL-C), that initially determine an endothelial dysfunction, an early step in CVD that contributes to plaque initiation and formation [3]. All CVD prevention guidelines recommend the use of statins to lower lipid levels although one third of statin users discontinue this therapy within one year from beginning. One of the reason of this non-adherence is represented to intolerance [4]. The recommended target level for total cholesterol (TOT-C) is less than 190 mg/dL in all patients, whereas the recommended level of LDL-C varies according to European Society of Cardiology/European Atherosclerosis Society (ESC/EAS) guidelines as follow: less than 115 mg/dL in subjects at moderate risk (≥ 1 ≤ 5%); less than 100 mg/dL in subjects at high risk (≥ 5 ≤ 10%); less than 70 mg/dL in subjects at very high total cardiovascular risk (≥10%). These targets should be achieved primarily by lifestyle changes represented to reduction in dietary sutured fat, use of functional foods enrich with phytosterols, reduction in excessive body weight and increase in habitual physical activity [5]. An alternative therapy included in the guidelines is the use of innovative nutritional strategies, based on consumption of specifically targeted “health” functional foods and/or dietary supplements—the so-called nutraceuticals—which can be used, together with dietary measurements, in alternatives or addition to lipid-lowering drugs so as not to have to increase their dose. The ESC/EAS guidelines mention phytosterols, soy, dietary fiber, policosanol, red yeast rice and berberine [5]. 

Aim of our study is to assess the effectiveness and safety of a dietetic supplementation of a new nutraceutical combination (NC) [berberine, red yeast rice (RYR) and monacolin K, policosanol, folic acid, coenzyme Q_10_ (CoQ_10_), asthaxantin, resveratrol, vitamin E, hydroxytyrosol, lycopene, and zinc] on lipid profile (TOT-C, HDL-C, and LDL-C, triglycerides), many inflammation biomarkers (hs-CRP, fibrinogen) and creatine phosphokinase (CPK) serum levels in patients with hypercholesterolemia.

## 2. Methods and Statistical Analysis

We enrolled 40 hypercholesterolemic patients (20 males, 20 females; 41 ± 7 years mean age; BMI 29.2 ± 1.3), non- smokers, who underwent hypolipemic treatment with NC (berberine [600 mg], RYR [200 mg], monacolin K [3 mg], policosanol [100 mg], folic acid [200 mcg], CoQ_10_ [50 mg], asthaxantin [0.5 mg], resveratrol [20 mg], hydroxytyrosol [20 mg], vitamin E [12 mg], lycopene [20 mg], and zinc [10 mg]).

Initial and final (after 12 weeks) screening included medical history, physical examination, and measurement of serum lipid profile (TOT-C, HDL-C, LDL-C, and TG), hepatic (GOT, GPT, γGT), and renal (serum creatinine and urea) functions, CPK levels and many inflammation biomarkers (hs-CRP and fibrinogen). At the screening visit, all patients were instructed to follow a normocaloric and hypolipidic diet during the study period. Were excluded to the study patients with intolerance to NC compounds, with renal and hepatic diseases, pregnant women, and patients treated with lipid-lowering drugs or substances. Adverse events were monitored throughout the study. All patients had to provide a written informed consent.

Data are presented as mean ± SD. The effect of treatment was analyzed by comparing the absolute changes from baseline values by means of *t*-Student test. Data were analyzed by using SPSS statistical software (SPSS Inc., Chicago, IL, USA).

## 3. Results

**Figure 1:**
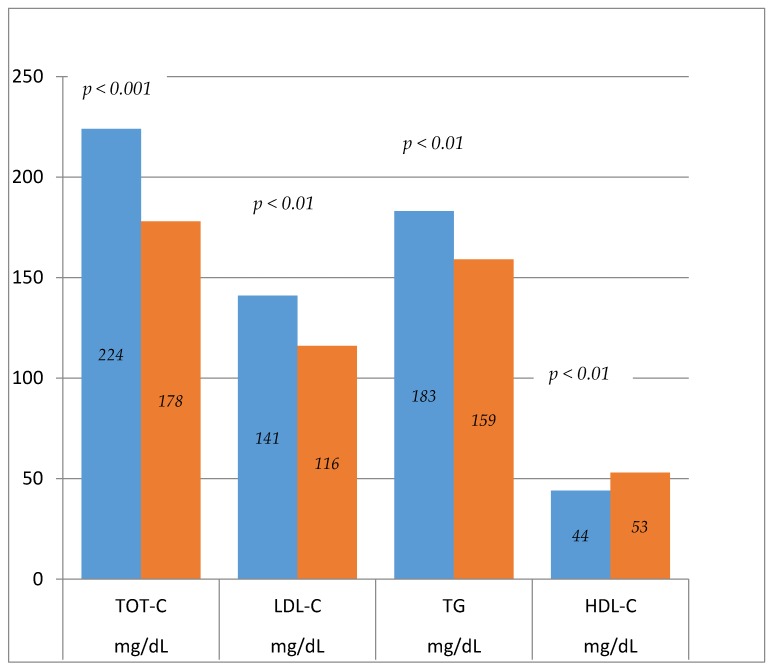
Lipid profile (pre-post treatment).

After 12 treatment weeks the comparison of changes respect to baseline values showed a significant reduction of TOT-C (224 ± 11.2 mg/dL vs. 178 ± 10.7; *p* < 0.001), LDL-C (141 ± 10.6 mg/dL vs. 116 + 10.1 mg/dL; *p* < 0.001), TG (183 ± 13 mg/dL vs. 159 ± 11.5 mg/dL; *p* < 0.01) (see Figure 1), serum inflammatory biomarkers as hs-CRP (2.24 ± 0.83 mg/dL vs. 1.76 ± 0.61 mg/dL; *p* < 0.01), fibrinogen (315 ± 43 mg/dL vs. 199 ± 41 mg/dL; *p* < 0.01) and a significantly increase of HDL-C (44 ± 7 mg/dL vs. 53 ± 7 mg/dL; *p* < 0.01) (see Figure 1). Hepatic and renal function and serum CPK were normal (see Table 1). No adverse events was reported.

## 4. Discussion

This study demonstrated that 12 weeks of treatment with a new NC significantly reduced TOT-C, LDL-C and TG values in subjects with mild-moderate hypercholesterolemia. This result was associated with a significant increase of HDL-C values and a significant reduction of inflammatory biomarkers (hs-CRP and fibrinogen). No adverse events were observed during the study period. 

The effects showed in the study are due to the important effects of NC. In particular RYR with monacolin K (3 mg) modulate the lipid profile by reducing TOT-C and LDL-C via “statin like mechanism” i.e., by competitively inhibiting HMG-CoA reductase [6]; the presence of monacolin K improve the affinity of this enzyme for the endogenous substrate [7]. 

Berberine, a natural alkaloid, may reduce expression of proprotein convertase subtilisin/kexin type 9 (PCSK9), and consequently lower LDL-C receptor degradation and increase liver low density lipoprotein receptor (LDL-R) expression with stimulation of hepatic uptake of plasma cholesterol, thus promoting clearance of LDL-C from the bloodstream [8]. Interestingly the mechanism of efficacy of berberine is independent to intracellular cholesterol plasma levels and is not diminished by other cholesterol-lowering substances. Additionally berberine reduces macrophage migration [9], and induced the expression of cholesterol efflux gene ATP-binding cassette transporter 1 (ABCA1), resulting in reduced intravascular accumulation of oxidized-LDL (ox-LDL) in human macrophages [10]. All these effects of berberine have been demonstrated in several randomized placebo-controlled trials involving patients with mild to moderate mixed hyperlipidemia, with or without type 2 diabetes mellitus and hyperlipemic patients with B or C cirrhosis. These studies have documented a significant reduction in LDL-C values (20–25%) and triglycerides (13–30%) [11]. 

CoQ_10_, an antioxidant present in many food sources, has an important role in the electron transport chain with the mitochondria. Given that CoQ_10_ and cholesterol synthesis share the same intermediate steps in their respective biosynthetic pathways, patients receiving statin treatment also experience a reduction in CoQ_10_ [12]. In addition, CoQ_10_ is essential for oxide reduction involved in ATP synthesis, prevent LDL peroxidation and represent one of the main antioxidant defenses of the human body involved [13]. 

Resveratrol (RES) is a natural phenol commonly found in the skin of grapes and is considered to be one of the key active compounds responsible for these cardiovascular protective effects. These effects are due to reduction of foam cells formation by inhibiting ox-LDL uptake as well as increasing cholesterol efflux in human THP-1 macrophages [14]. Several studies have found that higher average alcohol consumption (wine) reduce the incidence of CVD [15], CVD-event and all-cause mortality [16].

Lycopene is the carotenoid that gives tomatoes their bright red colour. These substance inhibits LDL oxidation in vitro [17] and might exert its anti-atherogenic effects by inhibiting de novo cholesterol synthesis. Several epidemiological studies have found an association between diets rich in lycopene and a reduced incidence of CVD, leading to several studies to further investigate its potential cardio-protective effects. [18,19]. 

Hydroxytyrosol represents the major anti-atherogenic polyphenol compounds in olive oil. Epidemiological studies have reported a correlation between increased levels of olive oil in the diet and a lower risk of developing atherosclerosis, and have suggested the use of hydroxytirosol as a nutraceutical for atherosclerosis. In particular these substance have demonstrated a reduced expression of the pro-inflammatory adhesion proteins vascular cell adhesion molecule 1 (VCAM-1) and intercellular adhesion molecule 1 (ICAM-1) in human umbilical vein endothelial cells (HUVEC) [20]. Many trials have investigated the healthy benefits of hydroxytyrosol supplementation. These studies have demonstrated a decrease in the ratio of T-CHOL and increase in HDL-C, with a reduction of markers of oxidative stress [21] such as IL-6 and CRP levels [22,23], IFN-γ [24], and ICAM-1 and monocyte number [25]. High hydroxytyrosol levels it is considered indicative of one of the major anti-atherogenic polyphenol compounds olive-oil.

Policosanol is a well-defined mixture of higher aliphatic primary alcohols isolated from sugar cane wax, with documented cholesterol-lowering effects proven for a dose range from 5–20 mg/day in patients with dyslipidemia [26] by increased hepatic LDL uptake and serum LDL catabolic rates, and the inhibition of hepatic cholesterol synthesis at the mevalonic acid stage of the pathway. A systematic review and meta-analysis of randomized controlled studies showed that policosanol was safe, well tolerated and effective for LDL reduction in patients with hyperlipidemia [27]. 

Vitamin E represent an important antioxidant considered as nutraceutical for its antioxidant and vasodilatory properties for the prevention of atherosclerosis [28]. Even if many studies have documented that vitamin E has not been proven to be consistently effective in long term prevention of CVD the anti-oxidative and vasodilatory properties of this vitamin are important for prevention of the atherosclerosis process. 

Zinc (Zn) is an agent with anti-oxidant, anti-inflammatory, anti-apoptotic and important activities into the regulation of lipid homeostasis. Many studies have demonstrated that zinc reduction resulted in iron overload, which thereby directly activated the conversion activity of stearoyl-CoA desaturase (SCD) with promotion of lipid biosynthesis and accumulation [29]. 

## 5. Conclusions

In this study our NC contains low doses of naturally occurring substances that exert complementary actions designed to prevent the formation of atherosclerotic plaques by significant and important lipid lowering effects after 12 treatment weeks. The important reduction of LDL-C values and inflammatory markers (h-PCR and fibrinogen) can be prevent the formation of atherosclerotic plaque, because this NC offer the opportunity to achieving significant reductions in C-LDL (−17.73%) and T-COL (−20.53%) levels, equivalent to what can be expected with low dose of statin therapy (lovastatin, simvastatin, pravastatin). For this reason this NC may be used in patients with mild hyperlipidemia intolerant to statin therapy, with chronic kidney disease, and liver disease.

A particular aspect of our study is represented by the low concentration of monacolin K (only 3 mg). Another important result of our study is the good safety and tolerability profile due to the intentional combination of low doses of its active ingredients. For these reasons this NC associated with dietary measurements, may be an excellent alternative for subjects with mild to moderate hyperlipidemia, in patients intolerant to statin, and for whom statins are not indicated (liver and kidney disease), and or in combination with other lipid-lowering agents in the attempt to avoid the untoward effects that are often associated with high dose pharmacological therapy. 

In conclusion this new NC, in addition to dietary measures, represent an excellent alternative for subjects with mild to moderate hyperlipidemia and for all dyslipidemic patients for whom statins are not indicated. The natural compounds included in our NC may be beneficial for the prevention or treatment of atherosclerosis, because they have anti-inflammatory properties and potential beneficial effects for to reducing CV risk. 

Our study has some limitations, namely a small sample size and short study duration. Further studies are required to fully evaluate the effectiveness and tolerability of this NC will lead to the identification of a novel treatment and preventive strategy in order to reduce lipid profile values and the global prevalence of CVD. 

## Figures and Tables

**Table 1:** Safety profile.

	T0 Pre-Treatment	T12 Post-Treatment
GOT (UI/L)	28	29
GPT (UI/L)	31	30
GGT (UI/L)	27	25
CPK (UI/L)	195	189
Crea (mg/dL)	0.97	0.94
Urea (mg/dL)	47	39
